# On the Origin of the Brain Semi‐Heavy Water Deuterium MR Signal Following Administration of Deuterated Metabolic Substrate: A Cautionary Tale

**DOI:** 10.1002/mrm.70347

**Published:** 2026-03-24

**Authors:** Joseph J. H. Ackerman, Xia Ge, Nick Rensing, Jeffrey J. Neil, Liu Lin Thio, Joel R. Garbow

**Affiliations:** ^1^ Departments of Radiology, Chemistry, Internal Medicine, and the Alvin J Siteman Cancer Center Washington University St. Louis Missouri USA; ^2^ Department of Radiology Washington University St. Louis Missouri USA; ^3^ Department of Neurology Washington University St. Louis Missouri USA; ^4^ Departments of Neurology and Radiology Washington University St. Louis Missouri USA; ^5^ Department of Radiology and the Alvin J Siteman Cancer Center Washington University St. Louis Missouri USA

**Keywords:** deuterium, DMI, fructose, HOD, mouse, MRI

## Abstract

**Purpose:**

To evaluate the extent to which the appearance of HOD in the brain following systemic administration of a deuterated substrate is due to local brain metabolism versus body metabolism.

**Methods:**

[6,6‐^2^H_2_]glucose, which is transported across the blood‐brain barrier (BBB), and [6,6‐^2^H_2_]fructose (Fruc), which does not cross the BBB, were administered to four mouse cohorts. Cohorts included wild‐type mice, glucose transporter deficiency mice, which have decreased brain glucose uptake from the blood, and littermate control mice. A separate wild‐type cohort received a 15‐μL intramuscular (leg) injection of D_2_O. Brain‐localized DMRS experiments employed the ISIS single‐voxel protocol at 11.74 T.

**Results:**

Following leg D_2_O injection, semi‐heavy water (HOD) appears within the brain in minutes, reaching steady state shortly thereafter. Body metabolism of [6,6‐^2^H_2_]fructose produces HOD that also appears in the brain within minutes following subcutaneous administration, with an initial rate (mM/min) substantially greater than following administration of [6,6‐^2^H_2_]glucose. Deuterated glucose from body metabolism of [6,6‐^2^H_2_]fructose also appears in the brain. The terminal rates for HOD appearance in the brain are indistinguishable for the four cohorts examined despite there being up to a five‐fold difference in brain concentration (mM) of deuterium‐labeled glucose.

**Conclusion:**

Body production of HOD dominates the initial increase of HOD in brain following administration of [6,6‐^2^H_2_]fructose and likely contributes significantly to the increase of HOD in brain following administration of [6,6‐^2^H_2_]glucose. Interpretation of HOD concentrations as representative of organ‐specific metabolism requires careful consideration of control experiments and assessment of HOD contributions from body metabolism of the administered deuterated substrate.

## 1 Introduction

Deuterium (^2^H) MR spectroscopy (DMRS) monitoring of metabolism in vivo, whether employing a single voxel or imaging protocol, continues to receive increasing attention [1, 2, 3, 4, 5, 6, 7, 8, 9, 10, 11, 12, 13, 14, 15, 16, 17, 18, 19, 20, 21, 22, 23, 24, 25] following seminal publications by University of Minnesota [26] and Yale University [27] research groups. DMRS generally shows substantial increases in semi‐heavy water (^1^HO^2^H = HOD) concentration following administration of deuterated substrate. Indeed, water, along with CO_2_, is a product of oxidative metabolism, and HOD often presents as the clearly dominant ^2^H resonance amplitude in DMRS studies. Given the strong HOD signal, recent reports [3, 6, 11, 13, 16] have employed metabolism‐driven water labeling to provide a biomarker of organ/tissue‐specific metabolic activity in vivo.

Water is a flow‐limited diffusible tracer [28, 29, 30, 31, 32, 33, 34, 35, 36], and many organs/tissues throughout the body may metabolize the administered substrate and produce HOD. Our recent research efforts have focused on brain DMRS with mouse models of epilepsy using deuterated glucose, acetate, and β‐hydroxybutyrate as substrates [37, 38, 39]. Given the proposal that HOD can serve as a biomarker of tissue‐specific metabolic activity, we wondered the extent to which the increase in HOD concentration could be ascribed *specifically* to brain metabolism versus overall body metabolism. This question begs the corollary: what is the timescale for HOD produced via non‐brain body metabolism of a deuterated substrate to transit to, and then be detectable in, the mouse brain by DMRS in vivo?

To explore this issue, we employed DMRS monitoring of brain metabolism in vivo following administration of [6,6‐^2^H_2_]glucose (Glc) or [6,6‐^2^H_2_]fructose (Fruc) in cohorts of wild‐type mice and mice deficient in the principal transporter of glucose across the blood brain barrier, Glut1 [40]. Under normal conditions, glucose is the near exclusive substrate for brain energy metabolism [41, 42, 43] and Glut1 is highly abundant in the blood brain barrier (BBB) [44, 45, 46, 47, 48, 49]. In contradistinction, Glut1 has very low affinity for fructose [50, 51] and fructose is generally not considered to be a significant metabolic substrate for brain [52]. While Glut5, the principal fructose transporter, has been found in the blood brain barrier [53], ^14^C‐labeled fructose injected into the right common carotid artery of the rat showed minimal accumulation in the brain [54], consistent with minimal BBB penetration found by others [55].

Because of the important role fructose plays in obesity, diabetes, metabolic syndrome, and non‐alcoholic steatohepatitis, its uptake and metabolism have long been studied (see for example, and references therein: [56, 57, 58, 59, 60, 61, 62, 63, 64]). Fructose is avidly extracted and metabolized in liver — 70% of fructose is metabolized by the liver in humans [57] — and to a lesser extent, in small intestine, kidney, skeletal muscle, and adipose tissue. Any increase in brain HOD detected following Fruc administration can, *in principle*, be ascribed predominantly to non‐brain body metabolism. Thus, our overall strategy was to compare the rate of HOD increase in brain (mM/min) as determined by DMRS following Fruc versus Glc administration, where brain HOD from Fruc can be ascribed to non‐brain, body (primarily liver) metabolism.

*In practice*, fructose metabolism produces dihydroxyacetone phosphate (DHAP) and glyceraldehyde, both of which can be phosphorylated to glyceraldehyde‐3‐phosphate (G3P) [65]. Both DHAP and G3P are intermediates of the gluconeogenic and glycolytic pathways, which can lead to glucose production with incorporation of a part of the fructose carbon structure ([64, 66] and citations therein). Indeed, blood glucose is known to increase after ingesting fructose and we provide evidence herein that the low‐level sugar signal observed in brain by DMRS following Fruc administration is not Fruc but predominantly glucose from liver fructose metabolism.

## 2 Methods

### 2.1 Deuterium MR Spectroscopy

DMRS was performed on an Agilent/Varian DirectDrive 11.74‐T small‐animal MRI scanner. The single‐voxel localization protocol employed the Image‐Selected In vivo Spectroscopy *(*ISIS) method [67] with outer volume suppression [68]. ^1^H MRI experiments to establish voxel placement coordinates and shim the magnetic field were performed using a 5‐cm ID homogeneous volume coil from ExtendMR LLC (Rancho Palos Verdes, CA). DMRS experiments employed a laboratory‐built 1.5‐cm circular surface coil. The eight‐step ISIS cycle included three frequency‐selective, 180‐degree hyperbolic secant adiabatic RF pulses for voxel selection and a non‐selective hard pulse for readout. Typical voxel dimensions were 3 × 6 × 5 mm^3^ (transaxial × sagittal × coronal). Other pulse sequence parameters included: 450 ms TR, 1500 Hz bandwidth, and 512 complex data points. For brain DMRS following administration of Gluc or Fruc, ^2^H free induction decays (FIDs) were acquired in repeated 5‐min data‐averaging time blocks. Two 5‐min acquisitions occurred prior to substrate administration (to quantify the natural‐abundance HOD signal amplitude as an internal concentration reference) and fourteen 5‐min acquisitions occurred immediately following substrate administration. For brain DMRS following intramuscular (leg) injection of 15‐μL D_2_O, ^2^H FIDs were acquired in repeated 2‐min data‐averaging time blocks. Six 2‐min acquisitions occurred prior to D_2_O administration, and twenty‐four 2‐min acquisitions occurred immediately following D_2_O administration, for a total of 60‐min of data acquisition.

### 2.2 Experimental Protocol

Protocols were approved by the Institutional Animal Care and Use Committee. Deuterated substrates were subcutaneously (sc) administered [24] to cohorts of wild‐type (WT) C57BL/6 mice, Glut1 haploinsufficiency (Glut1+/−) mice, a model of Glut1 deficiency syndrome (Glut1‐DS), and their littermate (Glut1+/+) controls. Details regarding these genetically engineered mice are provided below. Wild‐type mice received [6,6‐^2^H_2_]glucose or [6,6‐^2^H_2_]fructose, 2 g/kg, *N* = 5 each cohort. In total, these two cohorts contained ten female mice with age 45 ± 7 days (mean ± SD) and body weight 19.2 ± 1.2 g. To measure the rapid transit of water from the extracellular/extravascular space of tissue remote from brain to brain, a separate cohort of five female WT mice (age 45 ± 7 days; body weight 19.7 ± 0.9 g) received a 15‐μL intramuscular (leg, quadriceps femoris) injection of heavy water, ^2^H_2_O = D_2_O. Glut1+/− and Glut1+/+ cohorts, *N* = 5 each, were administered 2 g/kg [6,6‐^2^H_2_]fructose. In total, these two cohorts included seven female and three male mice with age 35 ± 5 days and body weight 18.7 ± 1.2 g. Commercial material sources were: C57BL/6 mice, Inotiv, formerly Envigo (Indianapolis, IN); Glut1+/− and Glut1+/− mice, Jackson Labs (Bar Harbor, ME), see below; [6,6‐^2^H_2_]glucose, Sigma Aldrich (St Louis, MO); [6,6‐^2^H_2_]fructose and D_2_O, Cambridge Isotope Laboratories (Andover, MA). Mice were anesthetized with isoflurane, Covetrus (Portland, ME) in O_2_, ∼3.0%–3.5% for induction and ∼1.0%–1.3% for maintenance. Respiration (90–120 breaths per minute) and core temperature (36.5°C–37.5°C) were monitored and controlled via adjustments to the isoflurane percentage and the temperature of warm air circulating through the magnet. Mice were not fasted and were scanned at various times throughout the normal workday.

### 2.3 Glucose Transporter 1 Deficiency Syndrome Mice

These studies used the *Tie2‐Cre; Slc2a1* ^*fl/+*^ mouse model of glucose transporter 1 deficiency syndrome (Glut1‐DS) [69]. These mice are haploinsufficient for Glut1 in brain endothelial cells because they have a floxed *Slc2a1* allele with *loxP* sites flanking exons 3–8 and an endothelial specific (*Tie2*) Cre driver. The *Tie2‐Cre; Slc2a1* ^*fl/+*^ mice were generated by mating mice homozygous for *loxP* sites flanking *Slc2a1* exons 3–8 (*Slc2a1* ^tm1.1Stma^/AbelJ) (Jackson Labs Strain 031871) with mice hemizygous for *Tie2‐Cre* (*Tek‐Cre*) (B6.Cg‐Tg(Tek‐cre)1Ywa/J) (Jackson Labs Strain 008863). Half the litter is haploinsufficient for *Slc2a1* in endothelial cells (*Tie2‐Cre; Slc2a1* ^*+/fl*^), the other half has normal brain endothelial levels (*Slc2a1* ^*+/fl*^) and serves as controls.

### 2.4 Data Analysis

DMRS monitors the uptake and metabolic transformation of deuterated substrates. Of specific relevance herein, the intracellular conversion in brain of deuterated glucose ([6,6‐^2^H_2_]glucose; Glc) to [3,3‐^2^H_2_]lactate (Lac) and Glx, the sum of a nominal 50/50 mix of [4,4‐^2^H_2_]glutamine + [4,4‐^2^H_2_]glutamate, was measured by single‐voxel brain DMRS. HOD is a byproduct of Glc metabolism following its conversion to pyruvate and entry into the mitochondrial matrix where, after oxidation to acetyl coenzyme A (acetyl‐CoA), it undergoes further oxidation, ultimately to water and CO_2_, in the tricarboxylic acid (TCA) cycle.

Liver metabolism of deuterated fructose similarly results in the production of HOD (see Zhang et al. [70], for metabolic pathway label tracking maps). Hepatic fructolysis bypasses the rate limiting step of glycolysis, conversion of fructose‐6‐phosphate to fructose‐1,6‐bis‐phosphate, and rapidly converts fructose into two triose phosphate intermediates that can ultimately undergo complete TCA oxidation in the mitochondrial matrix. Liver metabolism of [6,6‐^2^H_2_]fructose can also result in production and extravasation into the vasculature of deuterated glucose, which is then transported into the brain by Glut1, *vide infra*. To distinguish within the text the brain deuterated glucose signal originating from liver metabolism of deuterated fructose from that due to directly administered Glc, we label the brain ^2^H signal for glucose originating in the liver as Glc*.

The deuterium resonance amplitudes for Glc, Glc*, Glx, Lac, and HOD in brain were determined using Bayesian‐based time domain (FID) analysis software [71]. The natural‐abundance HOD signal amplitude, quantified before deuterated compounds were administered, served as a convenient internal standard (16.35 mM [72, 73] in St. Louis tap water). The amplitudes for Glc, Glc*, Glx, and Lac, proportional to species concentrations, were adjusted for relaxation effects and stoichiometry/label loss [7].

Findings for all cohorts are expressed as mean ± SD (*N* = 5). Statistical differences between cohorts were accepted at the 95% confidence limit, *p* < 0.05, via two‐way ANOVA with Tukey's multiple comparisons test (GraphPad Prism, version 10.6.1, Laguna Hills, CA).

## 3 Results

### 3.1 Labeled Water in Leg Muscle Appeared Quickly in Brain

**FIGURE 1:**
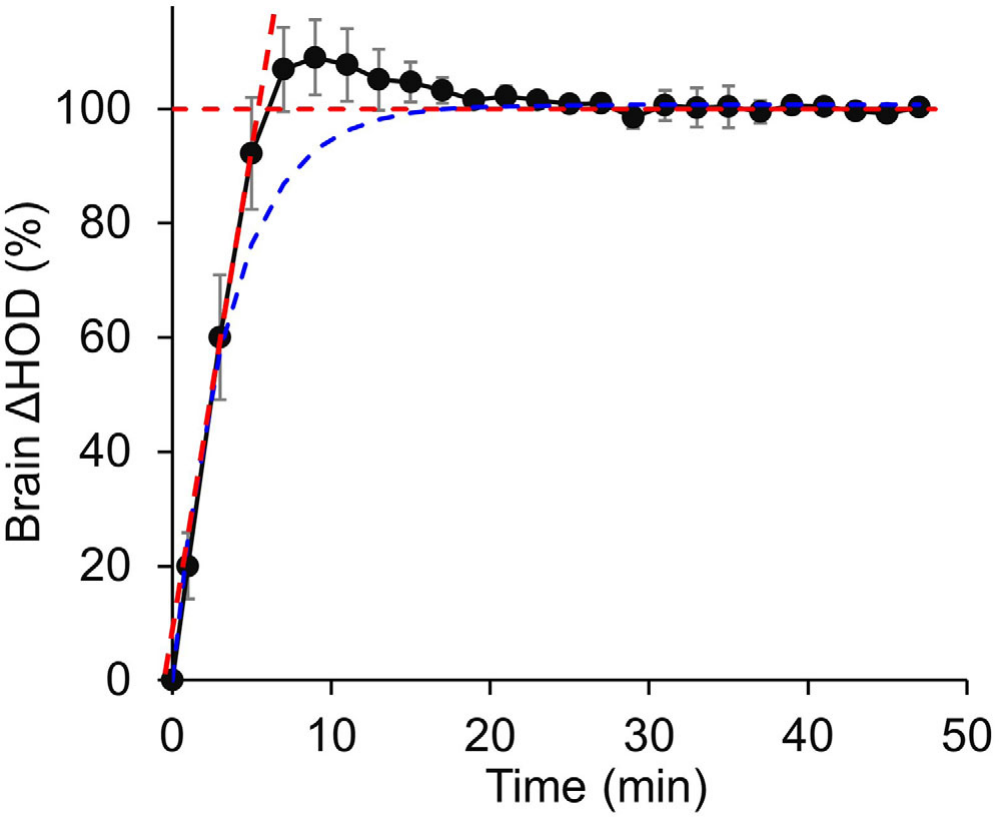
HOD appearance in brain after leg‐intramuscular injection. Timecourse for appearance of HOD in mouse brain following a 15‐μL D_2_O intramuscular injection into the mouse leg at time = 0; mean ± SD (*N* = 5). Data are plotted (abscissa) at the midpoint of each 2‐min acquisition time block. The red dashed line shows linear extrapolations of the data. The blue dashed line shows exponential modeling of the data excluding the steady‐state overshoot. Note, water ^1^H ⇄ ^2^H exchange is rapid on the timescale of this experiment.

In aqueous solution, the exchange lifetime for the hydrogen or deuterium atom in an individual water molecule is extremely short, tens of femtoseconds to picoseconds ([74, 75]; and references therein). Thus, modest amounts of D_2_O administered in vivo quickly become HOD (D_2_O + H_2_O ⇄ 2HOD). The normalized timecourse of the increase in brain HOD above natural‐abundance concentration, ∆HOD, following injection of 15‐μL D_2_O into leg muscle is shown in Figure 1. A remarkably rapid increase in brain HOD concentration during the initial 10 min post injection led to a steady‐state brain HOD concentration at ∼20 min. Indeed, the brain HOD concentration reached (and then overshot) the ultimate steady‐state level at ∼6 min post intramuscular injection.

### 3.2 A Brain Sugar ^2^H Signal Appeared Following Fruc Administration

**FIGURE 2:**
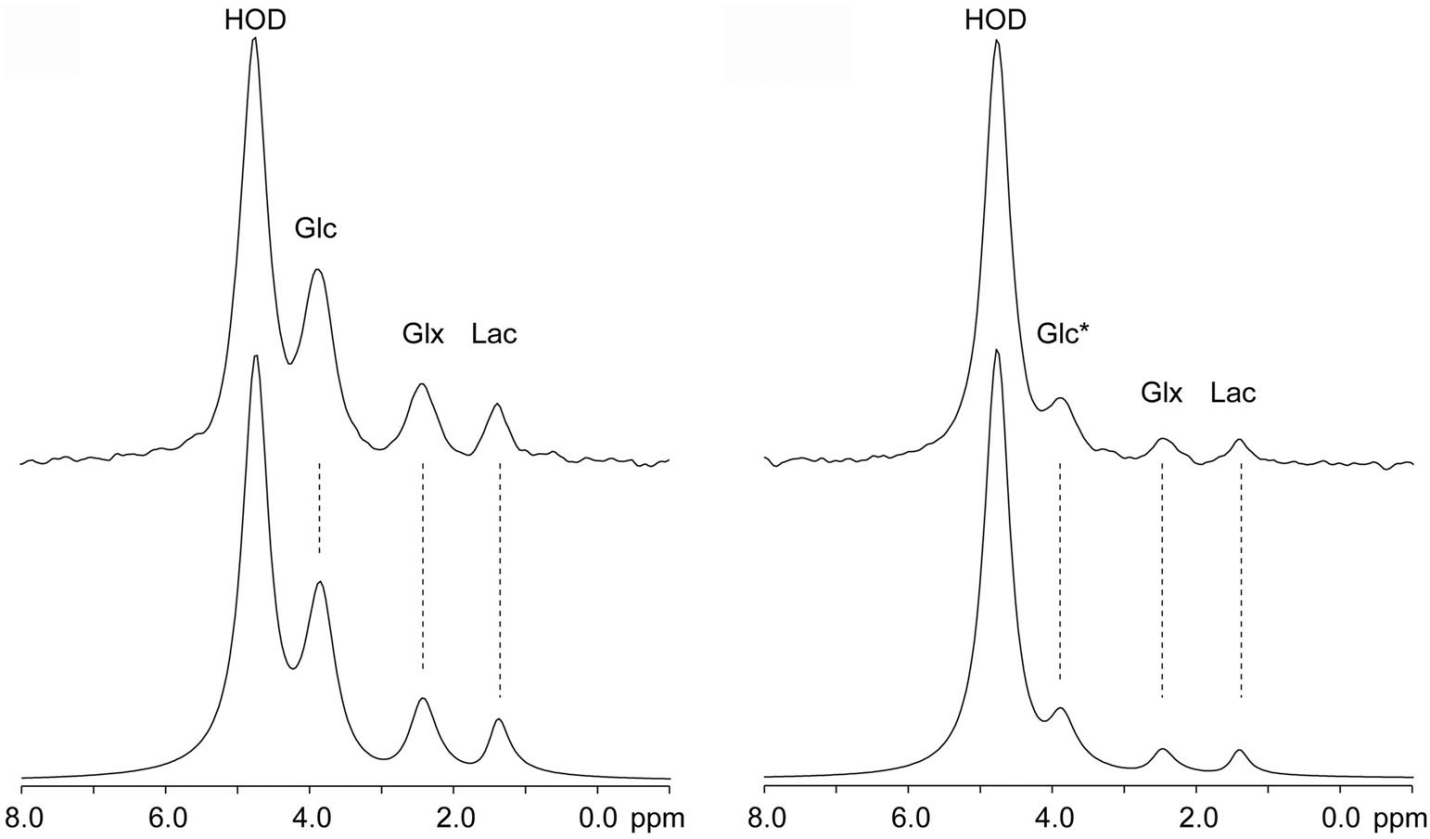
Mouse brain DMRS in vivo. Representative spectral data (top) and model (bottom) post sc administration of substrates Glc (left) and Fruc (right). Data are summed over the 70‐min timecourse. Labels are: HOD, semi‐heavy water; Glc, glucose (directly administered substrate); Glc*, glucose (from liver metabolism of fructose); Glx, sum of glutamate + glutamine; Lac, lactate. Spectral displays are scaled such that the HOD peak heights are the same in the left and right panels.

As the [6,6‐^1^H_2_] proton resonance frequencies of glucose and fructose are nearly identical [76, 77, 78], it follows that the [6,6‐^2^H_2_] deuterium resonance frequencies of Glc and Fruc will also be nearly identical [70, 79]. Therefore, considering the rather broad (∼15–30 Hz) DMRS resonance linewidths observed in mouse brain in vivo, the Glc and Fruc resonance lineshapes substantially overlapped, and Glc and Fruc could not be differentiated by chemical shift in the experiments described herein. Figure 2 shows representative mouse brain DMRS data, the result of summing fourteen 5‐min time blocks (70 min) for WT mice administered Glc (left panels) or Fruc (right panels).

The top panels show the Fourier‐transformed, frequency‐domain spectra after application of an exponential apodizing filter function leading to 10‐Hz line broadening. The bottom panels show the result of Bayesian signal modeling. Four resonances were present in each dataset: HOD, sugar (Glc or Glc*, *vide infra*), Glx, and Lac. The sugar signal (Glc*) in the mouse administered Fruc was substantially reduced in amplitude versus the mouse administered Glc, as were Glx and Lac.

### 3.3 The Sugar ^2^H Signal Observed in Mouse Brain Following Fruc Administration Was Glc*

**FIGURE 3:**
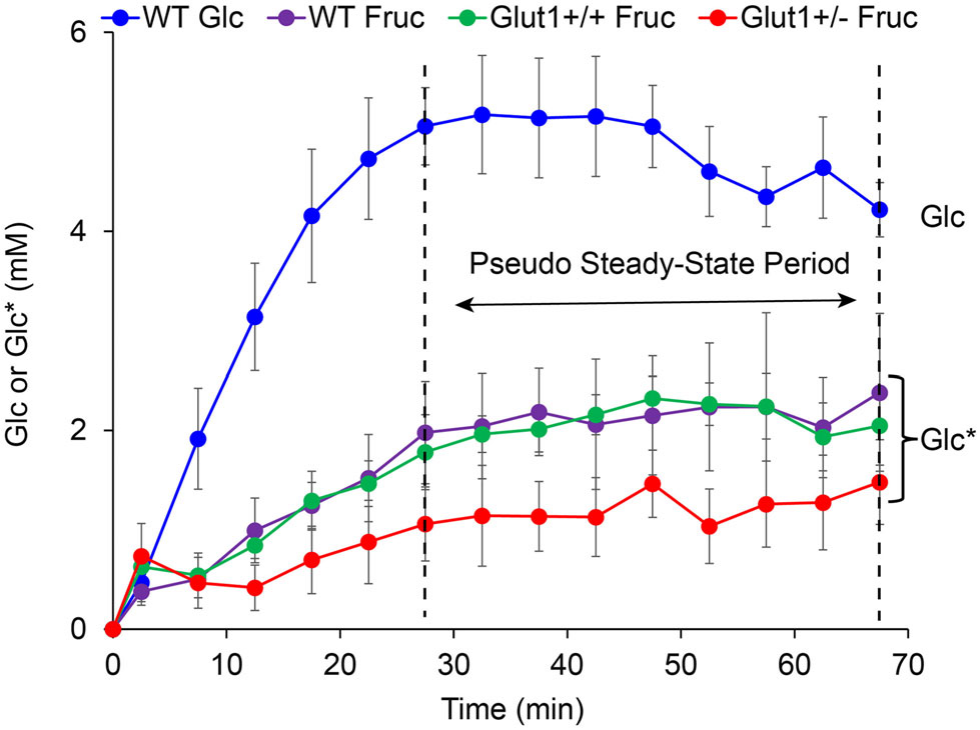
Mouse brain Glc or Glc* timecourse (mM, mean ± SD, *N* = 5). Blue symbols: WT cohort administered Glc; Purple symbols: WT cohort administered Fruc; Green symbols: Glut1+/+ cohort administered Fruc; and Red symbols: Glut1+/− cohort administered Fruc. Data are plotted at the midpoint of each 5‐min acquisition time block. The pseudo steady‐state period, during which Glc or Glc* brain concentrations remain relatively constant, occurs ∼25–70 min post sc administration of substrate (Glc or Fruc).

The DMRS sugar signal timecourses (mM, mean ± SD) are shown in Figure 3 for four mouse cohorts, in which each cohort is identified by “mouse‐type” and “substrate”: WT Glc, WT Fruc, Glut1+/+ Fruc, and Glut1+/− Fruc. Most strikingly, the brain ^2^H sugar signal from both WT mice and Glut1+/+ littermate‐control mice administered Fruc was substantially reduced versus WT mice administered Glc. As Fruc is not taken up into the brain, this signal is likely due to Glc generated from Fruc metabolism in the liver or other organs throughout the body. Importantly, during the Glc and Glc* pseudo‐steady‐state period (25–70 min post administration of Glc or Fruc), the ^2^H sugar signal amplitude in the Glut1+/− cohort was ∼50% lower than both the Glut1+/+ littermate‐control cohort and WT cohort administered Fruc.

More specifically, a 48% decrease was observed for the average sugar signal amplitude during the 25–70 min period post administration of Fruc between the Glut1+/+ versus Glut1+/− cohorts (1.83 ± 0.09 mM versus 0.96 ± 0.35 mM; *p* = 0.0007), providing strong evidence that the ^2^H sugar signal in mouse brain following Fruc administration was ^2^H labeled glucose (Glc*) originating from body metabolism of Fruc. Confirming evidence regarding the effect of Glut1 haploinsufficiency on brain Glc uptake comes from a separate ongoing study in which Glut1+/− mice administered 4.5 g/kg Glc (not Fruc) had a 41% lower ^2^H Glc signal amplitude than the Glut1+/+ littermate‐controls over the same period (data not shown).

### 3.4 The Brain ^2^H Labeled Semi‐Heavy Water Signal Was Dominated by Body Metabolism of Administered Substrate

**FIGURE 4:**
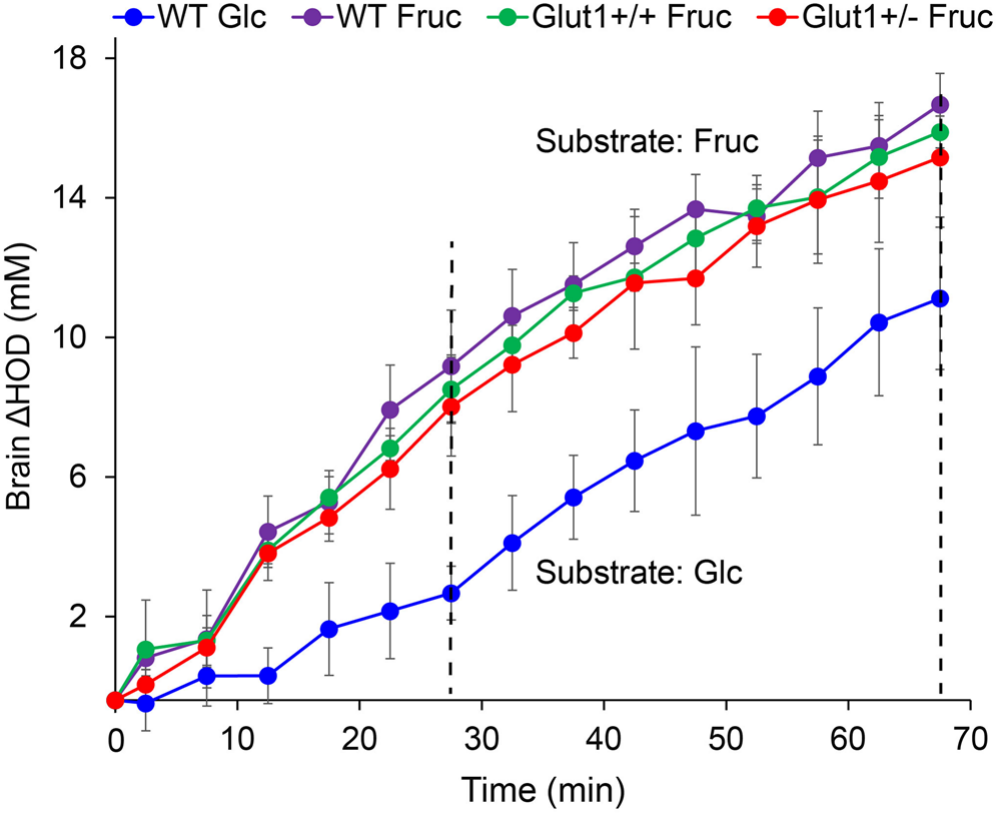
Mouse brain ∆HOD timecourse (mM, mean ± SD, *N* = 5). Blue symbols: WT cohort administered Glc; Purple symbols: WT cohort administered Fruc; Green symbols: Glut1+/+ cohort administered Fruc; and Red symbols: Glut1+/− cohort administered Fruc. Data are plotted at the midpoint of each 5‐min acquisition time block. The pseudo steady‐state period, during which Glc or Glc* brain concentrations remain relatively constant (Figure 3), occurs ∼25–70 min post sc administration of substrate (Glc or Fruc).

The ∆HOD timecourses (mM, mean ± SD, *N* = 5) for the four mouse cohorts (WT Glc, WT Fruc, Glut1+/+ Fruc, and Glut1+/− Fruc) are shown in Figure 4.

Two results were immediately evident. First, during the time period that Glc or Glc* are at pseudo‐steady‐state (25–70 min post administration of Glc or Fruc), the rate of brain ∆HOD increase (mM/min) was well modeled as constant (i.e., linear slope for ∆HOD vs. time) and is statistically the same (two‐way ANOVA; *p* > 0.4) across all four cohorts, despite a nearly five‐fold variation in brain Glc or Glc*. [Use of the period over which brain deuterated glucose levels (Glc, Glc*) achieve a quasi‐steady state provides a common reference metabolic timeframe with which to make comparison of the rate (mM/min) of HOD increase in brain across substrate (glucose, fructose) and genomic cohort (WT, Glut1+/+, and Glut1+/−)].

Second, early time points show a distinct lag in the brain ∆HOD increase for the WT mouse cohort administered Glc versus the three mouse cohorts administered Fruc (WT, Glut1+/+, and Glut1+/−). The HOD from body fructose metabolism rapidly transited to brain, resulting in a markedly increased slope of the brain ∆HOD timecourse relative to that observed immediately following administration of Glc as a substrate. Indeed, consistent with the timescale for appearance of HOD in brain following intramuscular (leg) injection of 15‐μL D_2_O, the brain HOD signal clearly increased above natural abundance level during the 10–15‐min data acquisition period post Fruc administration. As the TCA cycle is active within the mitochondria, this implies that the mitochondrial membrane(s) do not pose a significant hindrance to water efflux. Metabolism of Fruc to Glc, with subsequent production of HOD in brain and body organs (again via the TCA cycle), may also contribute to the observed brain HOD signal.

While the rate of HOD increase observed in brain from administered Glc does “catch up” with that from Fruc administration, the net amount of HOD (area under the curve) following Glc administration remains less than that following Fruc administration. This is presumably due to the regulatory (slow) phosphofructokinase‐1 (PFK‐1) step 3 in glycolysis. This regulatory step is absent in fructose metabolism, as is most clearly reflected in the early timecourse lag in HOD appearance in brain following Glc versus Fruc administration.

**FIGURE 5:**
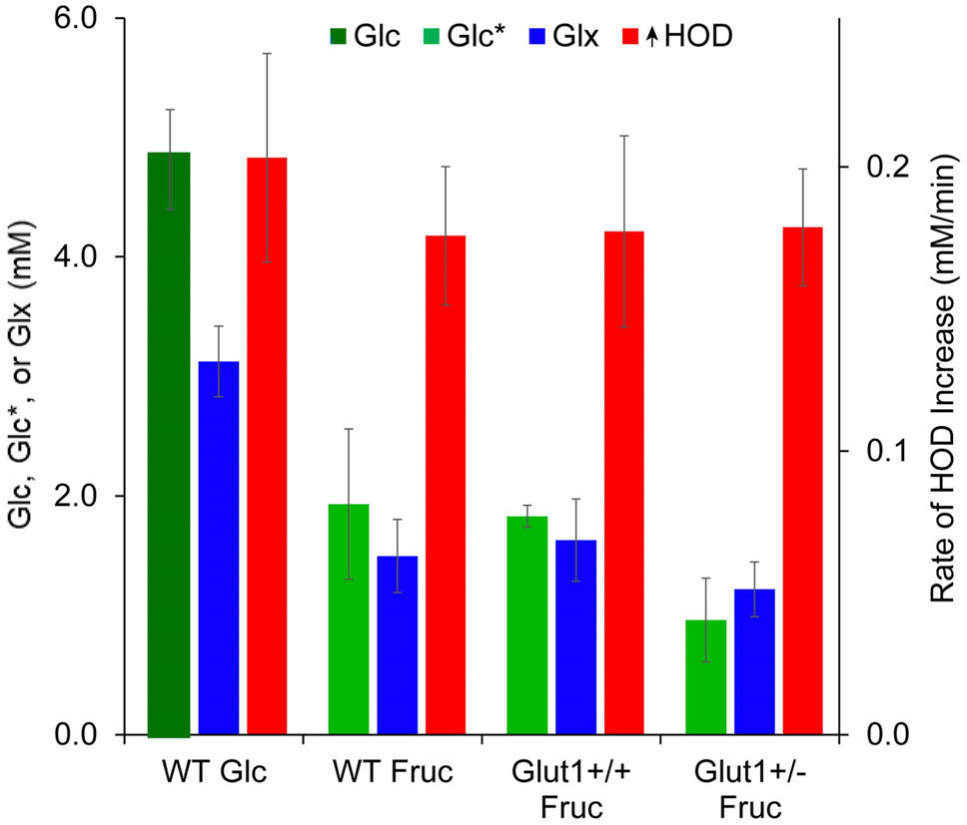
Rate of brain ∆HOD increase is independent of substrate. Left ordinate: average Glc, Glc*, and Glx concentrations (mM, mean ± SD) during the Glc or Glc* pseudo‐steady‐state period (i.e., the 25 to 70‐min period post sc administration of Glc or Fruc). Right ordinate: rate of brain ∆HOD increase (mM/min) during this period.

Figure 5 summarizes these findings in a bar graph display of average Glc, Glc*, and Glx concentrations (left ordinate, mM, mean ± SD) during the 25–70 min period post administration of Glc or Fruc and the rate of brain ∆HOD increase (right ordinate, mM/min) during this period. The Glx signal for Glut1+/− mice administered Fruc (cohort with lowest Glc*) was near the noise floor and its amplitude estimation was, thus, less certain than the other cohorts. Nevertheless, consistent with the Glc* level in the Glut1+/− cohort being ∼50% that in the Glut+/+ cohort, the brain Glx signal in the Glut1+/− cohort showed a trend (*p* = 0.056, unpaired *t*‐test; *p* = 0.17 two‐way ANOVA with Tukey's multiple comparisons test) toward an expected decrease compared to the Glut1+/+ cohort, again supporting identification of the sugar signal as Glc* (i.e., Glc* → pyruvate → acetyl‐CoA → TCA cycle → Glx).

## 4 Discussion

The DMRS HOD signal predominates in many protocols that administer deuterated substrates. It is well recognized that water is a flow‐limited diffusible tracer [28, 29, 30, 31, 32, 33, 34, 35, 36]. Indeed, labeled water has a rich history as a blood flow (perfusion) tracer [80, 81, 82]. Thus, an open question concerns the extent to which the DMRS HOD signal can be ascribed to tissue/organ specific metabolism versus whole body metabolism. Quantitatively, one would expect the answer to depend upon the subject under study; the tissue being monitored; the deuterated substrate, its molecular‐site‐specific labeling pattern and its route of administration; and the time resolution of the signal monitoring. We have approached the question qualitatively, focusing on DMRS of brain, an exemplar organ given its high metabolic rate, its preference for glucose as substrate, and the presence of a blood brain barrier that restricts entry of many substances.

The intramuscular D_2_O injection experiment confirmed that HOD initially present in the extracellular/extravascular space of tissue remote from brain appears in (transits to) brain in minutes, reaching steady state in brain shortly thereafter. However, this protocol does not mimic the production of HOD within the intracellular milieu and, importantly, within the mitochondria where membrane barriers might significantly impede transit of HOD to the tissue's extracellular/extravascular space. Nevertheless, the intramuscular injection protocol sets a lower limit, a few minutes, for the transit of remotely originating HOD to brain in mice.

The modest overshoot of the HOD signal before it decreases to a steady state level at ∼20 min post intramuscular (leg) injection of 15‐μL D_2_O is evidence of the flow‐limited nature of water diffusional distribution in tissue whereby HOD increases rapidly in brain because of the brain's relatively high blood flow before distributing throughout the body. Water is highly concentrated, 55.5 M, and even an intramuscular bolus of a volume as small as 15 μL into a ca. 20‐g mouse remains above the steady‐state whole‐body‐distributed HOD concentration at the time it reaches the brain. We have not explored the HOD transit characteristics following administration of smaller D_2_O volumes nor other entry points.

DMRS revealed a sugar ^2^H signal in the brains of mice administered Fruc, raising the obvious question of whether Fruc has entered the brain, an unexpected result. Based on the very similar ^1^H MR [6,6‐^1^H_2_] chemical shifts of protonated fructose and glucose [76, 77, 78], and given the relatively broad lineshapes of ^2^H resonances in vivo, we anticipated that [6,6‐^2^H_2_]‐labeled fructose and glucose would be indistinguishable by DMRS, as observed in recent liver DMRS studies of Glc and Fruc metabolism [70, 79]. Thus, we could not confirm the molecular identity of the brain sugar signal by DMRS alone.

However, compared to WT and Glut1+/+ littermate‐controls, Glut1+/− mice show a ∼50% reduction in brain sugar signal amplitude after administration of deuterated fructose. This reduction would only occur if the sugar was glucose, as Glut1 has low affinity for fructose [50, 51]. Confirming this effect, in a separate ongoing study, a ∼41% reduction in brain Glc was found comparing Glut1+/− mice to Glut1+/+ controls when both cohorts received 4.5‐g/kg Glc. Additionally, although not reaching statistical significance, comparison of brain Glx concentrations between Glut1+/+ and littermate‐control Glut1+/− mice administered Fruc showed a strong trend (*p* = 0.056) toward an expected decrease in Glx for the Glut1+/− cohort, which has the lowest sugar concentration, providing further evidence identifying the brain sugar signal following Fruc administration as Glc*. Thus, we conclude that the DMRS‐detected sugar signal in brain following Fruc administration was glucose (Glc*) produced by body metabolism of Fruc.

DMRS data acquisition for this study employed 5‐min signal averaging time blocks. Thus, the study is not sensitive to changes in HOD that occur over substantially shorter times. Nevertheless, as is clear from results shown in Figure 1 — the timecourse following direct D_2_O injection into mouse leg muscle — and in the initial portion of Figure 4 — the timecourse for appearance of HOD in mouse brain following subcutaneous administration of Fruc — HOD originating in tissue remote from the brain transits to and is detectable in brain within a few minutes. Given that HOD is a highly diffusible species that distributes throughout the body over a period of a few minutes in a blood‐flow dependent manner *irrespective of its point (source) of origin*, it follows that HOD originating in brain, e.g., from brain metabolism of Glc or Glc*, will exit brain and transit to other body tissues on the same time scale. It would be of interest to confirm the timescale for the complementary experiment, brain injection of D_2_O with monitoring of HOD in leg muscle.

Therefore, during the latter periods of the timecourse for HOD detected in brain (Figure 4), it is not possible a priori to assign a specific fraction of the HOD detected in brain as having *originated from brain metabolism* of deuterated glucose (Glc or Glc*) versus metabolism by other body tissues. However, we note that while mouse brain is well‐known to have a high resting metabolic output (∼6.4% of total), other tissues, especially liver, skeletal muscle, and heart (∼52.2, ∼13.0, ∼3.7%, respectively), contribute significantly to the total output [83]. Thus, it is likely that a substantial fraction of the HOD observed in brain in this study originated from the metabolism of deuterated fructose or glucose by tissues/organs other than brain. Control experiments monitoring the timecourse of appearance of HOD in other non‐brain tissues (e.g., muscle, liver) compared to the timecourse of appearance of HOD in brain would help to quantify the fraction of HOD in brain that can be attributed to brain metabolism versus body metabolism. More generally, control experiments monitoring the timecourse of HOD appearance in non‐targeted tissues following a specific substrate administration protocol (e.g., iv, sc, ip, oral) will be important if one wishes to use HOD appearance as a tissue‐specific marker of metabolic activity.

During the latter periods of the timecourse for HOD detected in brain (Figure 4), when the concentrations of Glc and Glc* were at pseudo steady state, 25–70 min post administration of Glc or Fruc, the increases in brain HOD signal amplitude were well modeled as linear in time. Further, these rates were statistically indistinguishable (*p* > 0.4) for the four cohorts examined despite an up to five‐fold difference in brain Glc or Glc* substrate levels (i.e., WT mice administered Glc versus Glut1+/− mice administered Fruc). This implies that during the pseudo steady‐state period for the mouse lines used herein under conditions of isoflurane anesthesia, the body's total TCA cycle output (rate of HOD production, mM/min) is equivalent whether the feedstock (substrate) is glucose or fructose.

As noted earlier, liver metabolism of fructose proceeds more rapidly than that of glucose, bypassing the rate limiting step of glycolysis. This metabolism quickly converts fructose into two triose phosphates that can, among other fates, undergo complete TCA oxidation in the mitochondrial matrix. This metabolic signature is clear in initial timecourse profiles for the appearance of HOD in brain (Figure 4) and is consistent with a recent DMRS report that contrasted the mouse liver timecourses of Fruc and Glc metabolism in vivo following bolus intravenous administration versus slow intravenous infusion [79]. In both cases, the increase in liver HOD under Fruc administration outpaced that under Glc administration. Given this difference in metabolic kinetics, it is likely that body metabolism of Fruc contributes throughout the timecourse to HOD observed in brain.

## 5 Conclusion

Body production of HOD contributes to the increase of HOD observed in brain following Fruc administration. While this is a somewhat specialized case in that fructose is not considered a significant substrate for brain but is rapidly metabolized in liver, we note that the ∆HOD signal is often dominant in DMRS monitoring of animal models and human subjects following administration of other deuterated metabolic substrates. Regarding the timecourse for HOD appearance in brain (Figure 4), there was a slower HOD increase immediately following Glc administration compared to that following Fruc administration even though glucose is the preferred substrate for brain metabolism. We caution that interpretation of ∆HOD concentrations as representative of local tissue metabolism requires careful control experiments and assessment of ∆HOD contributions from body metabolism of the administered deuterated substrate. Failure to account for production of HOD via metabolism of deuterated substrate by non‐targeted tissues would result in metabolic activity estimates for the targeted tissue that are artificially high, perhaps even dominated by off‐target‐tissue metabolism.

## Funding

This work was supported by the National Institutes of Health (P30CA091842, P50HD103525), the U.S. Department of Defense (HT94252510741), Dravet Syndrome Foundation, Engelhardt Family Foundation Innovation Fund, Foundation for Barnes‐Jewish Hospital, and the Mallinckrodt Institute of Radiology (MIR) Pilot Fund Program.

## Conflicts of Interest

The authors declare no conflicts of interest.

## Data Availability

The data that support the findings of this study are available from the corresponding author upon reasonable request.
